# Association Between Dietary Acid Load and Excess Weight in Adults: A Cross-Sectional Study

**DOI:** 10.3390/nu17223557

**Published:** 2025-11-14

**Authors:** Shurui Wang, Yisen Yang, Meijuan Lan, Zhaofeng Zhang, Qiang Tang

**Affiliations:** 1 Department of Nursing, The Second Affiliated Hospital, Zhejiang University School of Medicine, Hangzhou 310001, China; srphd@zju.edu.cn (S.W.); lanmj@zju.edu.cn (M.L.); 2 Department of Epidemiology and Biostatistics, Institute of Basic Medical Sciences Chinese Academy of Medical Sciences, School of Basic Medicine Peking Union Medical College, Beijing 100005, China; yyiyis@163.com; 3 School of Health Policy and Management, Chinese Academy of Medical Sciences & Peking Union Medical College, Beijing 100730, China; 4 Department of Nutrition and Food Hygiene, School of Public Health, Peking University, Beijing 100191, China; 5 Beijing’s Key Laboratory of Food Safety Toxicology Research and Evaluation, Beijing 100191, China; 6 The Second Affiliated Hospital Zhejiang University School of Medicine, Hangzhou 310001, China

**Keywords:** DAL, excess weight, PRAL, NEAP, China Health and Nutrition Survey

## Abstract

**Background**: Dietary acid load (DAL) influences acid–base balance and has been implicated in chronic metabolic disorders. However, its association with excess weight (EW; overweight/obesity) remains insufficiently studied, particularly in Chinese populations with unique dietary patterns. Clarifying this relationship is crucial for guiding targeted nutritional strategies aimed at reducing obesity and associated metabolic risks in China. **Methods**: This cross-sectional study analyzed data from 7758 adults in the China Health and Nutrition Survey (CHNS), a multistage, stratified cluster survey. Dietary intake was evaluated using three consecutive 24 h recalls, and DAL was calculated using potential renal acid load (PRAL) and net endogenous acid production (NEAP). The relationship between DAL and EW was analyzed using multivariable logistic regression, with additional insights gained from subgroup analyses and restricted cubic spline (RCS) methods. **Results**: The final analysis encompassed 7758 individuals, among whom 3072 (39.6%) were diagnosed with EW. After adjusting for all relevant factors, a higher DAL was found to be significantly associated with an increased risk of EW. Individuals in the highest tertile experienced a 27% increased risk associated with PRAL (OR = 1.27, 95% CI: 1.09–1.48, *p* = 0.002) and a 14% increased risk associated with NEAP (OR = 1.14, 95% CI: 1.01–1.29, *p* = 0.029), demonstrating a consistent linear trend (*p* < 0.001). Subgroup analyses showed that the positive association between DAL and EW was particularly evident in men (PRAL: OR = 1.40, 95% CI: 1.12–1.76; NEAP: OR = 1.46, 95%: 1.14–1.85) and in participants younger than 60 years (PRAL: OR = 1.32, 95%CI: 1.11–1.58). Importantly, the association remained significant among individuals without diabetes, hypertension, or heart disease (PRAL: OR = 1.26, 95% CI: 1.08–1.47). RCS analysis further confirmed a linear dose–response relationship between DAL and EW risk. **Conclusions**: This study establishes a significant dose–response relationship between higher DAL and increased risk of EW in Chinese adults. These findings underscore the potential of diets with lower acid load, particularly those rich in fruits and vegetables, as a strategic approach to mitigating the obesity epidemic.

## 1. Introduction

Based on the latest data from the World Obesity Federation’s report, approximately 2.6 billion individuals globally were affected by overweight or obesity in 2023, which constitutes nearly 38% of the total population [1,2]. Projections indicate that by 2035, this number is expected to exceed 4 billion, accounting for about 51% of the world’s population [3]. Of particular concern is the rapidly rising prevalence of obesity among children and adolescents. Between 2020 and 2035, the global obesity rate among boys is expected to increase from 10% to 20%, while among girls, it is projected to rise from 8% to 18% [4,5]. The escalating burden of excess weight and obesity poses a serious public health challenge worldwide, underscoring the urgent need for more proactive and comprehensive prevention and control strategies on a global scale.

In recent years, the study of obesity etiology has evolved beyond conventional energy balance models—which continue to hold partial explanatory value—to increasingly emphasize the influence of dietary composition and metabolic factors. Within this context, Dietary acid load (DAL) has emerged as a significant modulator of acid–base homeostasis and a potential contributor to the development of obesity. By altering systemic pH homeostasis and metabolic pathways, DAL may independently influence the pathophysiology of obesity [6]. DAL is commonly assessed using two indices: potential renal acid load (PRAL) and net endogenous acid production (NEAP). which estimate net acid generation from dietary intake and its impact on acid–base equilibrium [7,8]. Diets high in DAL—typically characterized by elevated intake of animal proteins and refined grains, alongside reduced consumption of fruits and vegetables—can induce chronic low-grade metabolic acidosis. Clinical evidence has linked elevated DAL with type 2 diabetes, cardiovascular disease, chronic inflammation, hypertension, chronic obstructive pulmonary disease (COPD), and hyperuricemia [9,10,11,12,13,14]. However, the relationship between DAL and excess weight (EW; overweight/obesity) has not been reported, especially within the Chinese population.

Potential mechanisms linking DAL to obesity may involve several indirect pathways. Chronic low-grade metabolic acidosis might suppress endocrine axes such as HPT and GH/IGF-1, reducing metabolic rate and promoting fat storage [15,16]. DAL could also contribute to insulin resistance via impaired insulin signaling and enhanced lipogenesis [17,18]. Additionally, low-fiber, high-DAL diets may disrupt gut microbiota, reduce SCFA production, and promote inflammation [19,20]. These mechanisms remain hypothetical and require further human validation.

Despite strong mechanistic and epidemiological evidence, the role of DAL in obesity remains debated. Experimental studies indicate that under severe pathological states—such as diabetic ketoacidosis or end-stage renal disease—acute metabolic acidosis activates the ubiquitin–proteasome pathway and muscle-specific E3 ubiquitin ligases, driving skeletal muscle proteolysis and lipolysis, which results in weight loss [15,16]. However, this catabolic response differs fundamentally from the effects of long-term diet-induced acidosis: the former reflects a compensatory hypermetabolic state, whereas the latter promotes energy storage and obesity through hormonal suppression, inflammation, and insulin resistance. At the population level, most studies, particularly from Western cohorts, support a positive association between DAL and obesity risk. Yet, this relationship has not been comprehensively investigated in large-scale representative samples of Chinese adults. Considering the ongoing Westernization of Chinese diets—marked by rising intake of animal products and refined grains and reduced consumption of fruits and vegetables—DAL levels in China are likely increasing. Therefore, research on the association between DAL and obesity in Chinese populations is urgently needed to strengthen theoretical understanding and guide the development of targeted nutritional interventions and public health strategies.

This study, based on data from the China Health and Nutrition Survey (CHNS), a multistage, stratified cluster survey aimed to investigate the association between DAL (assessed by PRAL and NEAP) and the risk of overweight and obesity among Chinese adults. Against the backdrop of the growing obesity epidemic in China and the poor long-term adherence to conventional weight management strategies, exploring the influence of DAL on obesity has substantial practical significance. Clarifying this relationship may provide novel scientific evidence for weight management and inform the development of feasible nutritional interventions—such as optimizing dietary patterns to improve acid–base balance—thereby offering a potential complementary approach to obesity prevention and treatment beyond calorie restriction.

## 2. Materials and Methods

### 2.1. Data Acquisition and Subjects

**Figure 1:**
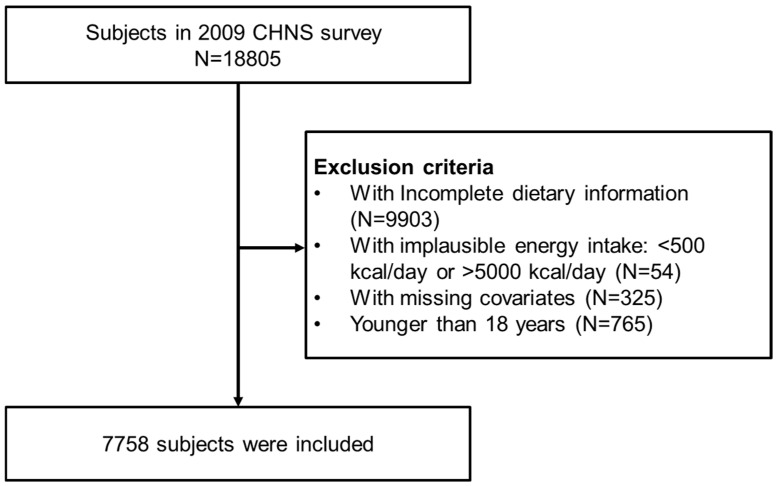
Screening flow.

Data were drawn from the 2009 wave of the CHNS, a wave selected for its comprehensive dietary and health examination data that were most consistent with our study objectives. The CHNS used a multistage, stratified cluster sampling method across nine provinces that were selected to capture the socioeconomic and geographic diversity of China, providing data with broad national representativeness for nutritional studies. A total of 18,805 participants provided demographic, health, dietary, and lifestyle information through questionnaires and physical examinations. Dietary intake was assessed by three consecutive 24 h recalls (two weekdays and one weekend day), with trained interviewers recording all food and condiment consumption. Nutrient intakes were calculated using the Chinese Food Composition Table, providing a reliable basis for estimating DAL (PRAL and NEAP). After excluding individuals with incomplete data, implausible energy intake (defined as a daily intake of less than 500 kcal or more than 5000 kcal, were excluded (N = 54)), a self-reported history of stroke, pregnancy, or age <18 years, 7758 adults remained for cross-sectional analysis (Figure 1).

### 2.2. Definition of EW

The Body Mass Index (BMI) serves as a metric for assessing body fat levels: underweight (BMI < 18.5), normal weight (18.5 ≤ BMI < 24), overweight (24 ≤ BMI < 28), and obese (BMI ≥ 28) [21]. Its calculation formula is as follows:

$$\mathrm{BMI}=\frac{Weight\left(kg\right)}{{Height(m)}^{2}}$$

EW was defined as a BMI ≥ 24.0 kg/m^2^, encompassing both the overweight (24.0 ≤ BMI < 28.0) and obese (BMI ≥ 28.0) categories according to Chinese criteria.

### 2.3. Dietary Acid Load Estimations

The average daily nutrient and energy intake was calculated based on three consecutive 24 h dietary recalls, using the Chinese Food Composition Tables. For the primary analysis, DAL (PRAL and NEAP) were calculated from absolute daily nutrient intakes (mEq/day) and were not energy-adjusted, using the following formulae [8,13,22]:

$$\begin{array}{cc} PRAL\left(\frac{mEq}{d}\right)= & 0.4888\times protein\left(\frac{g}{d}\right)+0.0366\times phosphorus\left(\frac{mg}{d}\right)\\ \; & -0.0205\times potassium\left(\frac{mg}{d}\right)-0.0125\times calcium\left(\frac{mg}{d}\right)\\ \; & -0.0263\times magnesium\left(\frac{mg}{d}\right) \end{array}$$

$$NEAP\left(\frac{mEq}{d}\right)=54.5\times protein\left(\frac{g}{d}\right)÷potassium\left(\frac{mg}{d}\right)-10.2$$

### 2.4. Other Variables

We systematically collected multidimensional data through standardized questionnaires, physical examinations, and laboratory tests. Demographic factors encompassed age, gender, marital status, living area, and level of education. Education level was categorized as primary school or less, middle school, high school, vocational school, or university or higher based on the highest completed schooling. Residence was classified as urban or rural per CHNS sampling criteria. Lifestyle factors were assessed based on smoking status and alcohol consumption, alcohol consumption was defined as a “Yes” response to “Do you drink alcohol?” in the CHNS questionnaire, identifying current drinkers. Smoking status was defined as a “Yes” response to “Do you smoke now?” identifying current smokers. Medical history was obtained from self-reports confirmed by physician diagnosis and covered diabetes, hypertension, heart disease, and fracture history. Anthropometric data comprised BMI, calculated from measured height and weight. Lab analyses encompassed blood lipid profiles [total cholesterol (TC), low-density lipoprotein cholesterol (LDL-C), high-density lipoprotein cholesterol (HDL-C), and triglycerides (TGs)], hyperuricemia based on gender-specific criteria, diabetes diagnosed through fasting blood sugar levels, hemoglobin A1c, or self-report, and estimated glomerular filtration rate (eGFR) calculated from creatinine levels. Nutritional intake was determined through three consecutive 24 h dietary logs, offering insights into daily consumption of macronutrients like carbohydrates, proteins, and fats.

### 2.5. Statistical Analysis

Data for continuous variables are expressed as mean ± SD or median (IQR), while categorical data are shown as counts (percentages). Statistical comparisons between groups were made using Student’s t-test, Mann–Whitney U test, or χ^2^ test, depending on the data type. DAL was evaluated using PRAL and NEAP, with participants categorized into tertiles based on their scores.

The relationship between DAL and EW was analyzed through multivariable logistic regression, and we constructed sequential models as follows: an unadjusted model (Model 1); a model adjusted for sociodemographic factors (Model 2: age, gender, marital status, education level, and urban/rural residence); a model further adjusted for comorbidities (Model 3: diabetes, hypertension, heart disease, and fracture history); and a fully adjusted model (Model 4) that included dietary components (total energy intake, fat, carbohydrate, and fiber). To mitigate multicollinearity, nutrients integral to the PRAL/NEAP formulae (protein, phosphorus, potassium, calcium, magnesium) were excluded from Model 4, which was confirmed by all variance inflation factor (VIF) values being below 2.5.

Stratified analyses by sex and comorbidities were conducted, with interaction effects evaluated via likelihood ratio tests. Non-linear associations were explored using restricted cubic spline (RCS) models, also adjusted for Model 4 variables, and non-linearity was tested similarly. The RCS models were fitted with 3 knots placed at the 10th, 50th, and 90th percentiles of the PRAL and NEAP distributions. The median value of each score was set as the reference point (OR = 1.0), the *p*-value for nonlinearity was formally tested.

All statistical analyses were conducted using R (version 4.2.3), with a significance level set at *p* < 0.05.

## 3. Results

### 3.1. Subject Characteristics

A total of 7758 individuals were ultimately chosen for analysis (Figure 1). Table 1 displays the overall clinical characteristics of all participants, with a mean age of 50.2 (±15.0) years. Of the 7758 participants, 4117 (53.0%) were females, and 3641 (47.0%) were males, and 84.9% of the participants were married. 22.7% of participants had a high school education or above. Regarding residency, the majority (2985, 38.5%) lived in urban areas. Nearly one-third of the participants consumed alcohol (32.7%) and 31.1% smoked cigarettes. More than 20% of participants had underlying diseases, including hypertension (1011, 13.0%), diabetes (215, 2.8%), heart disease (72, 0.9%), and fracture (361, 4.7%). The estimated nutrient intake yielded median PRAL and NEAP scores of 21.5 (13.5, 29.7) mEq/day, and 75.5 (63.4, 89.6) mEq/day, respectively.

Meanwhile, we found that the PRAL value was significantly higher in EW participants than that in the non-EW ones (22 (14.3, 30.5) vs. 21.0 (12.8, 29.2)). Similar results were observed for the NEAP value (76.4 (64.3, 9.9) vs. 75.1 (62.5, 89.3) Table 1). The nutrient intake between the two groups is compared in Table 2. We found that the EW participants tended to ingest significantly higher level of phosphorus (P), protein, and cholesterol compared to non-EW participants.

### 3.2. Association Between DAL and EW Risk

In all four multivariate-adjusted models, a significant positive association was observed between higher PRAL scores and increased EW risk (Table 3). Compared with the lowest tertile (T1), the odds ratios (ORs) in the fully adjusted model (Model 4) were 1.25 (95% CI: 1.11–1.42, *p* < 0.001) for T2 and 1.27 (95% CI: 1.09–1.47, *p* = 0.002) for T3. This association remained statistically significant across all models (*p* for trend < 0.001), and a significant dose–response relationship was consistently observed. When PRAL was analyzed as a continuous variable (per 10-unit increment), a robust positive association with EW risk was maintained throughout all adjustments (Table 3). In the fully adjusted model (Model 3), each 10-unit increase in PRAL corresponded to an OR of 1.11 (95% CI: 1.06–1.16, *p* < 0.001).

As shown in Table 4, the association between NEAP scores and EW risk was more modest. In the fully adjusted model (Model 4), the OR for the highest tertile (T3) was 1.15 (95% CI: 1.01–1.29, *p* = 0.029), while the association for T2 was not statistically significant (OR = 1.10, 95% CI: 0.98–1.24, *p* = 0.108). Nevertheless, a significant positive trend was identified across all models (*p* for trend = 0.012), suggesting a graded increase in risk with higher NEAP tertiles (Table 4). When evaluated as a continuous variable per 10-unit increase, NEAP also exhibited a significant yet weaker association with EW risk compared to PRAL. In the fully adjusted model, each 10-unit increase in NEAP was associated with an OR of 1.03 (95% CI: 1.01–1.06, *p* = 0.012).

Both PRAL and NEAP were positively associated with EW risk. PRAL demonstrated a stronger and more consistent association across tertiles and model adjustments, whereas NEAP showed a milder effect with partial loss of significance in the middle tertile, though a consistent positive trend remained. The continuous variable analyses further supported these findings, reinforcing the role of DAL as a potential risk factor for EW.

### 3.3. Subgroup Analysis of the Correlation Between DAL and EW

**Figure 2:**
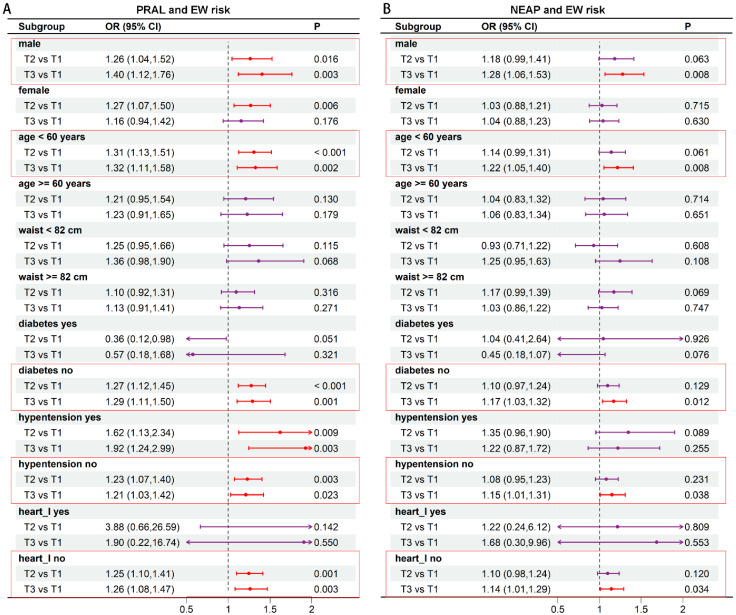
Scale represents Odds Ratio (OR). Stratified analysis of PRAL (**A**), NEAP (**B**) and EW risk. Scale represents Odds Ratio (OR)—Forest plot of the association between dietary intake and EW risk. The squares represent the point estimate of the odds ratio (OR), and the horizontal lines represent the 95% confidence intervals (CI). The size of the square may reflect the weight of each estimate. The vertical dashed line indicates the line of no effect (OR = 1). Associations are presented per increment of the specified nutrient.

To investigate potential heterogeneity, subgroup analyses were performed (Figure 2A,B). The findings from the PRAL analysis, stratified by gender, indicated that within the male cohort, individuals in T2 and T3 had a higher likelihood of experiencing EW compared to those in T1, after controlling for all confounding variables (OR 1.26, 95% CI: 1.04–1.52; OR 1.40, 95% CI: 1.12–1.76). Results stratified by age revealed a correlation between PRAL and the prevalence of EW. In the group aged under 60 years, the odds ratios (ORs) for T2 (1.31, 95% CI: 1.13–1.51) and T3 (1.32, 95% CI: 1.11–1.58) were significantly greater than those for T1. Additionally, in individuals free from diabetes, hypertension, or heart disease, a significant positive correlation was found between higher PRAL and increased prevalence of EW. The odds ratios (ORs) were 1.29 (95% CI: 1.11–1.50), 1.21 (95% CI: 1.03–1.42), and 1.26 (95% CI: 1.08–1.47) for tertile 3 (T3), as illustrated in Figure 2A. Additionally, we performed stratified analysis to examine the connection between NEAP and EW. Our findings revealed a notable association between NEAP levels and the risk of EW specifically in male participants, individuals aged over 60, and those without diabetes, hypertension, or heart disease. Notably, the risk of EW was substantially greater in the upper tertile than in the lower tertile (Figure 2B).

### 3.4. RCS Analysis of DAL and Risk of EW

**Figure 3:**
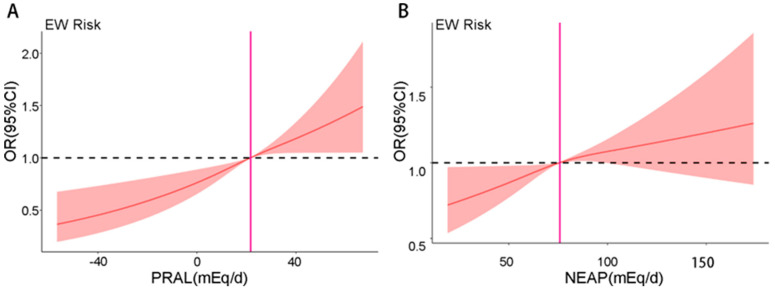
RCS analysis of the association between PRAL (**A**), NEAP (**B**) and Risk of EW. (**A**) Association between PRAL and EW risk with adjusted ORs; (**B**) Association between NEAP and EW risk with adjusted ORs.

**Figure 4:**
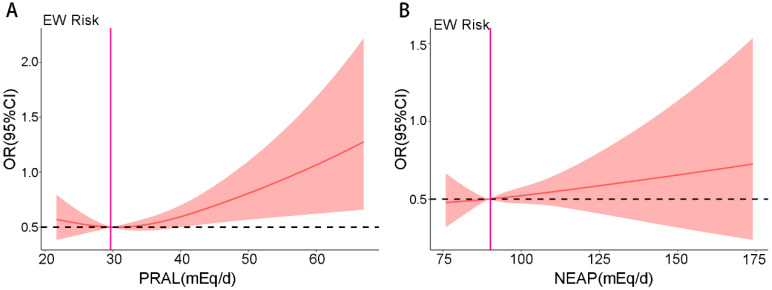
RCS analysis of the relationship between DAL and EW risk. (**A**) Subgroup analysis using the PRAL cut-off (>21.67 mEq/d) for adjusted ORs; (**B**) Subgroup analysis based on the NEAP cut-off (>75.83 mEq/d) for adjusted ORs.

We conducted an RCS analysis to visualize the association between DAL and EW risk. The result showed that the OR of EW possesses an upward trend with the increase in the PRAL and NEAP score, which indicated a significant linear relationship between PRAL/NEAP and EW (Figure 3A,B). To further elucidate this association and enable a clear stratified analysis, we dichotomized the cohort into ‘lower’ and ‘higher’ DAL groups. For this purpose, we used the median values of PRAL (21.67 mEq/d) and NEAP (75.83 mEq/d) as clinically relevant cut-offs. This stratification (Figure 4A,B) confirmed a strong and clear linear association, underscoring the heightened EW risk among individuals with higher acid load scores (Figure 4A,B).

### 3.5. The Association Between Dietary Intake and the Risk of EW

**Figure 5:**
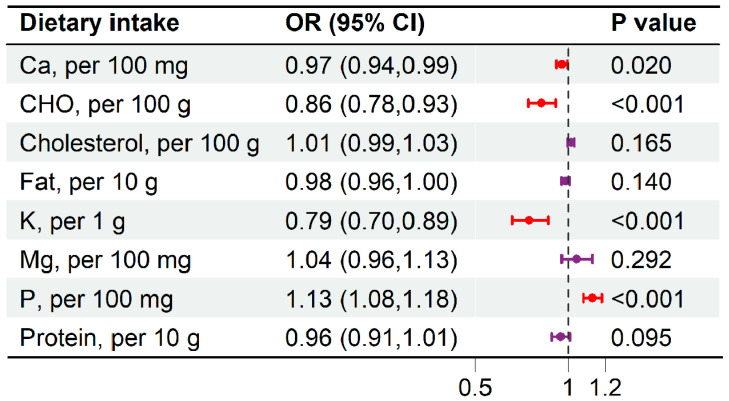
Scale represents Odds Ratio (OR)—Forest plot of the association between dietary intake and EW risk. The squares represent the point estimate of the odds ratio (OR), and the horizontal lines represent the 95% confidence intervals (CI). The size of the square may reflect the weight of each estimate. The vertical dashed line indicates the line of no effect (OR = 1). Associations are presented per increment of the specified nutrient.

An energy-adjusted model (intake per 1000 kcal) was applied in a separate analysis to assess the association between dietary intake and the risk of EW. Each 100 mg increase in phosphorus intake was associated with a 13% higher risk of EW (OR: 1.13, 95% CI: 1.08–1.18, *p* < 0.001). In contrast, higher intakes of calcium, carbohydrates (CHO), and potassium were linked to a reduced risk: each 100 mg increase in calcium intake corresponded to a 3% lower risk (OR: 0.97, 95% CI: 0.94–0.99, *p* = 0.020); each 100 g increase in CHO intake to a 14% lower risk (OR: 0.86, 95% CI: 0.79–0.93, *p* < 0.001); and each 1 g increase in potassium intake to a 21% lower risk (OR: 0.79, 95% CI: 0.70–0.89, *p* < 0.001) (Figure 5).

## 4. Discussion

This study is the first nationwide analysis of the link between DAL and EW in the Chinese population. Based on a multistage, stratified cluster survey, it provides data with broad national representativeness. Results show that higher PRAL and NEAP scores—both key indicators of DAL—are significantly associated with an increased EW risk. Subgroup analyses revealed that the association was robust in males and those under 60 years of age. A linear correlation between acid load scores and EW was further confirmed by RCS analysis. By clarifying the role of DAL in weight development, this study addresses an important research gap and offers meaningful implications for weight management and obesity prevention strategies.

The significance of acid–base balance is increasingly recognized in nutritional medicine and related disciplines [22]. It is now well established that dietary choices play a crucial role in modulating an individual’s acid–base balance, and that DAL can be intentionally modified through nutritional interventions [23,24]. Estimating a diet’s acidogenic potential often relies on PRAL and NEAP calculations [25,26]. Accumulating evidence indicates that DAL exerts considerable influence on various health outcomes, including cardiovascular diseases, cerebrovascular disorders, vascular conditions, and even aging [9,14,27,28,29]. For example, Mozaffari and colleagues found that elevated DAL correlates with a heightened risk of cardiovascular disease and metabolic syndrome among women [30]. Similarly, a study in a southern Chinese population found that elevated PRAL scores were positively correlated with hypertension and hyperuricemia [9]. Additionally, multiple studies suggest that high DAL is associated with impaired renal function [31], including findings by Kanazawa indicating that elevated NEAP predicts higher prevalence of albuminuria and microalbuminuria [32], and research by Toba et al. showing that increased NEAP may contribute to decreased glomerular filtration rate [33]. Nevertheless, the relationship between DAL and endocrine disorders—particularly EW—remains underexplored.

Based on our research, this appears to be the pioneering investigation into how DAL relates to EW among Chinese individuals. Our results demonstrate a significant positive correlation between both PRAL and NEAP scores and the incidence of EW. Subgroup analyses indicated that this relationship remained consistent across strata defined by gender, age, and hypertension, with reduced heterogeneity observed in these subgroups. These findings align with a previous cross-sectional study conducted among 203 EW adolescents in Iran, which reported that higher DAL levels were associated with an increased likelihood of metabolically unhealthy obesity [34]. That study further revealed that the association with NEAP was more pronounced in overweight than in obese individuals, and also linked higher PRAL and NEAP values with elevated fasting blood glucose and triglyceride levels. Together, this supporting evidence reinforces the validity and accuracy of our findings.

While our primary analysis demonstrated a robust positive association between DAL and EW risk, stratified analyses revealed several non-significant associations within specific subgroups. These findings warrant careful interpretation. The lack of statistical significance in subgroups such as older adults (age ≥ 60 years), females (for the T3 vs. T1 comparison of NEAP), individuals with larger waist circumference, and those with pre-existing diabetes or heart disease is most likely attributable to limited statistical power. These subgroups inherently had smaller sample sizes or a lower number of EW events, reducing the study’s ability to detect a true effect even if one exists, as reflected in the wider confidence intervals observed [35]. Alternatively, these results may indicate a genuine attenuation of the DAL effect in these populations. In older adults or individuals with established metabolic disorders, the influence of dietary acid load on EW risk might be overshadowed by more dominant pathophysiological factors, such as age-related decline in renal function, chronic inflammation, or severe insulin resistance [36]. Furthermore, we cannot rule out the possibility of effect measure modification or unmeasured residual confounding specific to these subgroups. Therefore, the non-significant associations should not be interpreted as definitive evidence of no effect but rather as findings that highlight the need for confirmation in future studies with larger, dedicated cohort sizes for these specific subpopulations.

The significant positive association between DAL, as indicated by PRAL and NEAP scores, and EW risk observed in this national study may be explained by several interrelated biological mechanisms. Chronic high acid intake can induce low-grade metabolic acidosis, which has been shown to impair insulin signaling and promote insulin resistance—a fundamental driver of adipogenesis and obesity [37]. Concomitantly, acid–base imbalance disrupts key hormonal regulators of appetite and metabolism; specifically, it may suppress leptin secretion (increasing hunger) while stimulating cortisol release (promoting central fat deposition), collectively leading to sustained positive energy balance [23]. Furthermore, the physiological effort to buffer excess acid through mobilization of bicarbonate and skeletal calcium not only increases energy expenditure but also triggers compensatory hyperphagia, ultimately favoring net weight gain [38]. Finally, acidogenic diets—typically characterized by high animal protein and refined grains with low fruit and vegetable content—can alter gut microbiota composition, reducing beneficial short-chain fatty acid production and thereby impairing metabolic and inflammatory homeostasis [6]. These mechanisms collectively operate in concert, suggesting that high DAL may contribute to EW through multifaceted metabolic, endocrine, and microbial pathways, highlighting the importance of dietary acid–base balance in public health strategies for obesity prevention.

This study, based on energy-adjusted models, revealed heterogeneous effects of dietary nutrients on the risk of EW. Higher phosphorus intake significantly increased EW risk, likely due to the widespread use of phosphorus additives in processed foods, which can disrupt calcium–phosphorus balance, induce insulin resistance, and reflect unhealthy dietary patterns. In contrast, calcium, carbohydrates, and potassium showed protective associations. Calcium may exert benefits by modulating lipid metabolism, while the protective effect of carbohydrates likely underscores the importance of carbohydrate quality, such as sources rich in whole grains and dietary fiber, commonly found in traditional Chinese diets, rather than refined carbohydrates [39]. Potassium intake demonstrated the strongest benefit, potentially through improving electrolyte balance and enhancing insulin sensitivity [40]. Collectively, these findings highlight that EW prevention should extend beyond total energy control to focus on dietary quality and composition, emphasizing reduced consumption of phosphorus-rich processed foods and increased intake of natural foods rich in calcium, potassium, and high-quality carbohydrates such as whole grains and legumes.

The current study has several limitations. First, it is a cross-sectional analysis reliant on data from the CHNS-2009, of which the cross-sectional nature restricts our ability to draw definitive conclusions about cause-and-effect relationships. Subsequent investigations should consider prospective and longitudinal designs to validate and confirm these observations. Secondly, we employ PRAL and NEAP to calculate the overall DAL. Although these algorithms are widely applied, they are indirect algorithms because it does not directly measure the body’s acid–base status; instead, they infer its impact on the kidneys by assessing the acidic and alkaline components in the diet. Additionally, PRAL does not account for the digestive absorption efficiency of nutrients in the gastrointestinal tract, which is another crucial aspect of how diet affects acid–base balance. Thirdly, although we adjusted for a wide array of confounders, important variables such as precise physical activity metrics, sodium intake, and processed food consumption were not available in the dataset and could not be included. To quantify the potential impact of such unmeasured confounding, we calculated E-values. The E-value for the association between the highest PRAL tertile and EW (OR = 1.27) was 1.54, and for the highest NEAP tertile (OR = 1.14) it was 1.37. This indicates that an unmeasured confounder would need to be associated with both high DAL and EW by risk ratios of at least 1.54 and 1.37, respectively, to fully explain away the observed associations, which suggests a reasonable degree of robustness to residual confounding. Fourth, our study was unable to independently examine the association between DAL and obesity (BMI ≥ 28 kg/m^2^) due to a limited sample size in this subgroup, which warrants investigation in future larger-scale studies. Lastly, the use of BMI categories, while clinically meaningful, may not capture the full continuous relationship between dietary acid load and body weight. Despite these limitations, the study has strengths. First, it is the first multicenter, nationally representative investigation. Moreover, even after considering a wide array of confounding variables, both dietary and non-dietary, the significant positive association between DAL and EW persisted, enhancing the trustworthiness and representativeness of the findings.

## 5. Conclusions

In summary, this study identifies a significant dose–response association between DAL and EW, both with and without adjustment for covariates. The findings revealed that higher scores of PRAL/NEAP were positively correlated with the prevalence of EW. Furthermore, RCS analysis confirmed a clear linear relationship between the risk of EW and PRAL/NEAP scores. These findings suggest that a diet lower in acid load may be a promising strategy for weight management. However, future longitudinal or interventional studies are required to establish causality before any clinical or public health recommendations can be formulated.

## Figures and Tables

**Table 1:** Subject characteristics of the study participants.

Variable	Overall N = 7758	None EW N = 4686	EW N = 3072	*p*
Age	50.2 (15.0)	49.5 (15.9)	51.3 (13.3)	<0.001
Gender				0.745
Female	4117 (53%)	2494 (53%)	1623 (53%)	
Male	3641 (47%)	2192 (47%)	1449 (47%)	
Education level				<0.001
Primary	3423 (44%)	2059 (44%)	1364 (44%)	
Middle	2577 (33%)	1558 (33%)	1019 (33%)	
High	887 (11%)	530 (11%)	357 (12%)	
Vocational	518 (6.7%)	330 (7.0%)	188 (6.1%)	
University	353 (4.6%)	209 (4.5%)	144 (4.7%)	
Marry				<0.001
Single	450 (5.8%)	363 (7.7%)	87 (2.8%)	
Married	6586 (85%)	3880 (83%)	2706 (88%)	
Other	722 (9.3%)	443 (9.5%)	279 (9.1%)	
Location				<0.001
Rural	4773 (62%)	3031 (65%)	1742 (57%)	
Urban	2985 (38%)	1655 (35%)	1330 (43%)	
Alcohol	2538 (33%)	1488 (32%)	1050 (34%)	0.028
Smoking	2409 (31%)	1512 (32%)	897 (29%)	0.005
Hip	94.0 (89.0, 99.1)	90.6 (87.0, 94.4)	100.0 (96.0, 104.0)	<0.001
Waist	82.0 (75.0, 89.9)	77.0 (72.0, 82.5)	90.0 (85.0, 96.0)	<0.001
PRAL	21.5 (13.5, 29.7)	21.0 (12.8, 29.2)	22.0 (14.3, 30.5)	<0.001
NEAP	75.5 (63.4, 89.6)	75.1 (62.5, 89.3)	76.4 (64.3, 89.9)	0.004
Diabetes	215 (2.8%)	70 (1.5%)	145 (4.7%)	<0.001
Hypertension	1011 (13%)	419 (9.0%)	592 (19%)	<0.001
Heart Disease	72 (0.9%)	32 (0.7%)	40 (1.3%)	0.007
Fraction history	361 (4.7%)	198 (4.2%)	163 (5.3%)	0.031

Data are presented as mean (SD), n (%), or median (IQR). *p*-values were derived from Student’s *t*-test, Mann–Whitney U test, or χ^2^ test, as appropriate. Abbreviations: EW, Excess Weight; PRAL, Potential Renal Acid Load; NEAP, Net Endogenous Acid Production.

**Table 2:** Energy and nutrient intakes (Energy-adjusted, per 1000 kcal).

Nutrients	Overall (N = 7758)	None-EW (N = 4686)	EW (N = 3072)	*p*
Total Energy (kcal)	1770.3 (1467.3, 2108.3)	1762.7 (1459.3, 2097.0)	1795.3 (1493.7, 2127.0)	0.030
Protein (g/1000 kcal)	37.3 (32.6, 43.9)	37.2 (32.4, 43.8)	37.6 (33.0, 44.1)	0.014
Fat (g/1000 kcal)	25.2 (16.0, 34.1)	25.0 (15.9, 34.0)	25.5 (16.1, 34.3)	0.202
Cholesterol (mg/1000 kcal)	198.4 (95.7, 318.5)	196.0 (91.6, 317.3)	204.0 (103.3, 320.4)	0.062
Carbohydrates (g/1000 kcal)	153.1 (131.6, 176.2)	154.0 (132.8, 176.5)	151.7 (130.5, 175.4)	0.063
Fiber (g/1000 kcal)	5.5 (4.2, 6.3)	5.5 (4.2, 7.3)	4.6 (4.2, 5.3)	<0.001
Ca (mg/1000 kcal)	191.3 (149.1, 257.1)	191.7 (149.3, 258.7)	190.2 (148.6, 255.4)	0.439
P (mg/1000 kcal)	536.2 (480.1, 602.4)	532.6 (476.4, 601.2)	541.9 (487.2, 604.9)	<0.001
K (mg/1000 kcal)	945.5 (798.1, 1120.9)	948.7 (798.4, 1123.7)	937.4 (797.5, 1109.0)	0.261
Mg (mg/1000 kcal)	154.1 (132.5, 181.5)	154.1 (132.4, 181.3)	154.2 (132.6, 182.0)	0.404

Data are presented as median (IQR). *p*-values were derived from the Mann–Whitney U test.

**Table 3:** Association between DAL (PRAL) and the Risk of EW.

PRAL Exposure	Model 1		Model 2		Model 3		Model 4	
	OR (95% CI)	*p*	OR (95% CI)	*p*	OR (95% CI)	*p*	OR (95% CI)	*p*
Tertile 1 (Ref)	1	-	1	-	1	-	1	-
Tertile 2	1.23 (1.10, 1.37)	<0.001	1.19 (1.06, 1.33)	0.003	1.20 (1.07, 1.35)	0.002	1.25 (1.11, 1.42)	<0.001
Tertile 3	1.24 (1.11, 1.39)	<0.001	1.17 (1.04, 1.31)	0.008	1.16 (1.03, 1.31)	0.011	1.27 (1.09, 1.47)	0.002
***p* for trend**	0.001		0.001		0.001		0.001	
**Per 10-unit increase**	1.09 (1.06, 1.13)	<0.001	1.07 (1.03, 1.11)	<0.001	1.07 (1.03, 1.11)	<0.001	1.11 (1.06, 1.16)	<0.001

Model 1 was unadjusted; Model 2 was adjusted for sociodemographic factors (age, gender, marital status, education level, and urban/rural residence); Model 3 was further adjusted for comorbidities (diabetes, hypertension, heart disease, and fracture history); and Model 4 was fully adjusted for dietary components (total energy intake, fat, carbohydrate, and fiber) in addition to all previous covariates. The continuous PRAL exposure is presented as per 10-unit increase (10 mEq/day).

**Table 4:** Association between DAL (NEAP) and the Risk of EW.

NEAP Exposure	Model 1		Model 2		Model 3		Model 4	
	OR (95% CI)	*p*	OR (95% CI)	*p*	OR (95% CI)	*p*	OR (95% CI)	*p*
Tertile 1 (Ref)	1	-	1	-	1	-	1	-
Tertile 2	1.11 (0.99, 1.24)	0.063	1.09 (0.98, 1.23)	0.125	1.10 (0.98, 1.23)	0.117	1.10 (0.98, 1.24)	0.108
Tertile 3	1.16 (1.03, 1.29)	0.011	1.13 (1.01, 1.26)	0.038	1.12 (1.00, 1.26)	0.05	1.15 (1.01, 1.29)	0.029
***p* for trend**	0.012		0.012		0.012		0.012	
**Per 10-unit increase**	1.03 (1.01, 1.06)	0.004	1.03 (1.00, 1.05)	0.021	1.03 (1.00, 1.05)	0.026	1.03 (1.01, 1.06)	0.012

Model 1 was unadjusted; Model 2 was adjusted for sociodemographic factors (age, gender, marital status, education level, and urban/rural residence); Model 3 was further adjusted for comorbidities (diabetes, hypertension, heart disease, and fracture history); and Model 4 was fully adjusted for dietary components (total energy intake, fat, carbohydrate, and fiber) in addition to all previous covariates. The continuous PRAL exposure is presented as per 10-unit increase (10 mEq/day).

## Data Availability

This study analyzed publicly available datasets (https://www.cpc.unc.edu/projects/china/data).

## Abbreviations

The following abbreviations are used in this manuscript:

DAL	Dietary acid load
EW	Excess weight
PRAL	Potential renal acid load
NEAP	Net endogenous acid production
RCS	Restricted cubic spline
CHNS	China Health and Nutrition Survey
BMI	Body Mass Index
TC	Total cholesterol
LDL-C	Low-density lipoprotein cholesterol
HDL-C	High-density lipoprotein cholesterol
TGs	Triglycerides
eGFR	Estimated glomerular filtration rate
